# The Influence of Social Crowding on Consumers’ Preference for Green Products

**DOI:** 10.3389/fpsyg.2022.832869

**Published:** 2022-04-25

**Authors:** Feng Wenting, Wang Lijia, Gao Cuixin

**Affiliations:** ^1^Gemmological Institute, Research Center for Psychological and Health Science, China University of Geosciences, Wuhan, China; ^2^Research Center for Psychological and Health Sciences, Institute of Education, China University of Geosciences, Wuhan, China; ^3^Research Center for Psychological and Health Sciences, Institute of Advanced Studies, China University of Geosciences, Wuhan, China

**Keywords:** social crowding, green products, safety needs, extraversion-introversion personality trait, consumers’ preference

## Abstract

With the increasingly crowded shopping environment, social crowding has become an important factor that affects consumers’ psychology and behavior. However, the impact of social crowding on consumers’ preference for green products hasn’t been focused on. Therefore, the aim of this paper is to empirically investigate the influence of social crowding on consumers’ preference for green products. With four studies, the present research examines how social crowding influences consumers’ preferences and uncovers the underlying psychological mechanism. The research shows that consumers prefer green products more under the condition of high social crowding than low, and safety needs mediate the impact of social crowding on green products preference. However, the impact of social crowding on the preference for products is only significant in green products. It also demonstrates the moderating effect of introversion-extraversion personality traits between social crowding and green products preference. For extraverted consumers, social crowding won’t affect their preference for green products, while for introverted consumers, social crowding is more likely to increase their preference for green products. This study contributes to marketing research by proposing and testing a new mechanism that underlies social crowding.

## Introduction

Considering the serious environmental effects, protecting the environment has become one of the main focuses of the world over the years, and fortunately, society has engaged in actions to deal with the environmental problems. Environmental concerns and values have influenced a person’s willingness to be environmentally friendly (Kautish and Sharma, 2019a; Kautish et al., 2020). Consumers with environmental awareness are paying more attention to their purchasing behaviors. For example, consumers often choose to switch from conventional products to sustainable or “green” products (Griskevicius et al., 2010).

Green products refer to products that use fewer resources, have lower impacts and risks to the environment, and prevent waste generation (Evangelia and Grigoris, 2019). They are less or not at all harmful for the environment in comparison to a substitute of the same product category (Moser, 2016). Hence, under this situation, green products seem to be a key point in the protracted battle of the preservation of the environment. However, although many enterprises actively guide consumers to participate in green consumption, the market performance of green products is not ideal. Sales of green products appear to be trending downward, resulting in insufficient green consumption (Gleim et al., 2013). Therefore, the analysis of the relevant factors affecting consumers to buy green products has become a key issue to improve green consumption.

Social crowding is a common social situation, such as crowded supermarkets and shopping malls in big cities. It is also an important research variable that affects consumers’ psychology and behavior (Jang et al., 2015). It’s a common phenomenon that people will be in a crowded social environment when buying green products. According to market performance, consumers seem more willing to buy green products in the situation of high social crowding. For example, in crowded auto shows, new energy, and other environmentally friendly vehicles are often the main theme (Sohu, 2020). Similarly, in the crowded market during the holiday, some low-carbon and green products are especially favored by consumers (Xu, 2010). The more crowded the store, the better green products sell (Li, 2014). So, what impact will social crowding have on consumers buying green products? What is its internal mechanism?

Scholars have made a good exploration on the antecedents affecting the purchase intention of green products from individual and contextual perspective. Previous literature mainly focus on individual factors influencing the decision and perception of green consumption (Kautish and Sharma, 2019a). For example, demographic variables (Chekima et al., 2016; Sreen et al., 2018), consumer personality (Lu et al., 2015), past consumption experience (Kumar et al., 2017), pro-social self-concept (Johnson et al., 2018), a desire for status (Griskevicius et al., 2010), environmental awareness (Kautish et al., 2019), political orientation (Watkins et al., 2016), terminal value and instrumental value (Kautish and Sharma, 2018, 2019b; Kautish et al., 2020), green skepticism (Kreczmańska-Gigol and Gigol, 2022), cosmopolitanism, global self-identity (Khare and Kautish, 2020) and so on. Few studies explore the effect of contextual factors on consumers’ buying of green products. For example, social environment (Johnstone and Hooper, 2016), specific social groups (Salazar et al., 2012), green advertising and green brand image (Alamsyah et al., 2020), perceived store accessibility (Chang and Watchravesringkan, 2018), availability and ease of purchase (Grimmer et al., 2016), and store-related attributes of eco-fashion (Chan and Wong, 2012), etc. affect green purchase behavior.

To sum up, existing studies mostly discuss relevant factors from the individual level, social relationship perspective, the characteristic of brand and store, but lack of the exploration on social crowding. However, social crowding is a common and important environmental factor on green purchase behavior. The current studies ignore the impact of social crowding on consumers’ preference for green products, which can’t provide an integrative perspective on green purchase behavior. Therefore, this paper empirically investigates the influence of social crowding on consumers’ preference for green products, which aims to make up for the research limitation and enriches the research on environmental factors in the field of green consumption.

Based on the theory of needs for safety (Maslow, 1943), the current research explores the impact of social crowding on the preference for green products. It contains four studies. Study 1 found that social crowding will increase consumers’ preference for green products. Study 2 demonstrated that safety needs mediate the relationship between social crowding and consumers’ preference for green products. Study 3 clarified the impact of social crowding on the preference for products is only significant in green products. Study 4 proved the positive relationship between social crowding and consumers’ preference for green products is more likely to occur for introverted consumers rather than extraverted consumers.

## Literature Review and Hypothesis Development

### Social Crowding

Social crowding is defined as a large group of people gathered together such that the likelihood of an individual’s personal space being violated is significantly increased (Maeng et al., 2013). It refers to the closed and restricted feeling experienced from the high crowd density (Li et al., 2009). Under social crowding, the subjective psychological feeling caused by the fact that the space provided by the environment is insufficient to meet the individual’s needs due to a large number of people in a unit area (Stokols, 1972; Park and Zhang, 2019). Hence, perceived crowding is a subjective state of psychological stress that arises from a situation of scarce space (Stokols, 1972; Blut and Iyer, 2019), which essentially leads to the threat of invasion of one’s territory (Anninou et al., 2018).

In recent years, with the improvement of people’s consumption level, the shopping environment has become more and more crowded, and researchers have paid more attention to the study of consumers’ preference for products under the crowded situation. Previous research has demonstrated that people will assess both higher prices and a greater willingness to pay for products presented in less crowded contexts (O’Guinn et al., 2015), and an increase in perceived crowding in a retail store can decrease the level of satisfaction that shoppers have with the store (Machleit et al., 2000). However, when consumers feel that their personal space is violated and experience a need to express their individuality, in this case, they are more likely to choose products that distinguish them from others (Xu et al., 2012). Moreover, Maeng et al. (2013) argue that being socially crowded leads to both a greater preference for safety-related choice options and to increased accessibility to safety-related words through invoking an avoidance motivation. It can be seen that social crowding is an important factor affecting consumers’ purchasing behavior. However, in the field of green consumption, the impact of social crowding on consumers’ preference for green products remains to be further studied.

### Green Products

Green products are described in various literature. A “green product” is referred to a product designed to minimize its environmental impacts during its whole life-cycle (Roberts, 1996). Parker et al. (2009) point out that a green product may represent a product that does not harm the natural environment, an organic product, or one that includes no artificial components, and also may be production-based or associated with sustainable corporate activity. Green products can be positioned as both sustainable and effective to environmental causes (Lin and Chang, 2012). In other words, a green product is a product that is friendly to human beings, the environment, and society. Green products can also deliver certain information. When a product is labeled “organic,” it indicates to consumers that it has been produced in accordance with organic standards throughout production, handling, processing, and marketing stages, and certified by a duly constituted certification body or authority (Rao and Lenka, 2020).

Moreover, in addition to the utility obtained directly from a purchased good, green consumers can also receive psychological benefits from buying an environmentally friendly product (Hartmann and Apaolaza-Ibáñez, 2012). The purchase of green products can be characterized as pro-social behavior (Johnson et al., 2018) and consumers may experience a feeling of wellbeing from acting in such a way, that is, personal satisfaction by contributing to the improvement of the common good environment (Kaida and Kaida, 2016). Beyond this, consumers can also obtain self-expressive benefits from green consumer behaviors (Policarpo and Aguiar, 2019). Thus, the consumption of green products in public may deliver an individual benefit because it allows consumers to present a positive imagine of themselves to others (Trudel, 2019).

### Green Products and Safety

Safety refers to the premonition of possible physical or psychological danger or risk, as well as the individual’s strong feeling in dealing with things, mainly manifested as a sense of certainty and control (Maslow, 1943). Prior research has shown the sense of safety can affect consumers’ marketing behaviors. For example, the lower the consumers’ sense of food safety, the less their motivation to consume food (Wansink et al., 2012); When the need for safety is likely salient, consumers are likely to show an increased preference for luxury brands (Han et al., 2019). However, although the sense of safety is an important factor affecting consumers’ psychology and behavior, the existing research does not demonstrate the role of the sense of safety in the field of green consumption. Hence, in the context of this study, the researchers purpose that green product has many attributes that can activate consumers’ safety.

The whole life cycle of green products, including production, use, and waste disposal, can provide people with a kind of safety value. Specifically, green is a subset of sustainability and is usually linked to reducing environmental impacts (Taubitz, 2010). In terms of the development of the green product, it aims to prevent pollution from the beginning through product design and innovation by using non-toxic compounds or biodegradable materials during the production process in order to protect the environment and to improve energy efficiency (Xie et al., 2019; Khare and Kautish, 2020). Moreover, compared with regular products with unsustainable materials (Kautish et al., 2021), green products tend to use biodegradable, non-toxic ingredients and recyclable packaging (Lin and Chang, 2012). The non-renewable resource use of the green product is minimized, toxic materials are avoided and renewable resource use takes place in accordance with their rate of replenishment (Sdrolia and Zarotiadis, 2019). Thus, the production process of green products is more friendly to the environment and can provide a safer living environment for human beings.

Moreover, green products have a kind of safety value that consumers feel that the consumption of the product is harmless and free from synthetic chemicals (Jan et al., 2019). Take green food for instance, people are more willing to choose organic food for its healthier and safer than conventional food (Magnusson et al., 2003). Typical environmental attributes about green consumption include recyclability, recycled content, fuel efficiency, toxic content reduction, and emission-related performance (Magnusson et al., 2003). Hence, green products hold the potential to aggregate long-term benefits, reduce consumer stress and ameliorate customer environmental responsibilities while maintaining positive qualities (de Medeiros et al., 2014). That’s to say, green products can provide consumers with a more secure consumption choice in the process of use. Therefore, using green products can provide people with a sense of safety.

After use, green products strive to protect or enhance the environment by reducing or eliminating pollution and waste (de Medeiros et al., 2014). Generally speaking, it is of economic and environmental interest to recycle the value remaining in used products (Pigosso et al., 2010). For instance, recovered co-products give more environmental gains than do avoided landfill (Blengini et al., 2012). Additionally, economic savings and reduction in energy consumption can be gained by recycling manufacturing and end-of-life product waste (Gaustad et al., 2018). Except for the fact that environmentally sound remanufacturing or recycling processes can be easily applied, the environmental impact of the disposal or incineration is minimal when finally discarded (Sdrolia and Zarotiadis, 2019). In this case, considering the benefit that green products bring to the environment in the stage of waste disposal, the researchers suggest that green products can make people feel safer.

In a word, through the efficient use of resources, low impacts and risks to the environment, and waste generation prevention since their conception stage (Albino et al., 2009), green products can provide safety value psychologically for consumers in the production, use and waste disposal stages.

Besides, the symbolic value represented by green products enables consumers to better integrate into the group, be accepted by others, gain a sense of belonging to the group, protect the stability of their interpersonal relationships, alleviate the threat of social exclusion, hence activate consumers’ sense of safety. Specifically, decisions to buy may be related to social goals and needs because buying certain products makes people feel connected to others (Salazar et al., 2012). Consumers buy goods not only to satisfy a variety of non-social needs like nourishment and shelter but also to establish and maintain social relationships (Salazar et al., 2012). Similarly, consumers are seen not only as independent decision-makers driven by internal attitudes and values but rather as shaped by their social interactions and the influence of others, and their behavior is shaped with a frame of reference produced by the social groups to which each individual belongs (Aagerup and Nilsson, 2016).

Symbolic products are social tools, which individuals use to communicate and construct their identity to their social networks (Chiesa and Dekker, 2022). For instance, green products can be a considerable source of social status that some consumers use to communicate identity to their peers (Griskevicius et al., 2010) and when deciding whether to buy green products, people tend to look to others (Salazar et al., 2012), suggesting that green product is the way of people to gain social acceptance. Moreover, consumers will be inclined to buy pro-social items to signal information about being pro-social members of society (Johnson et al., 2018). However, people who behave pro-socially can effectively reduce and alleviate the threat of social exclusion (DeWall and Richman, 2011). For example, consumers who feel socially excluded have been shown to engage in helping behaviors and charitable donations (Johnson et al., 2018). Hence, the researchers claim that green products allow consumers to integrate into the group to gain social acceptance and avoid social exclusion.

People will hunger for affectionate relations with people in general, namely, for a place in a group to get a sense of belonging (Maslow, 1943; Gopalan and Brady, 2020). The need to belong is defined as the desire to form and maintain close, lasting relationships with some other individuals (Baumeister and Leary, 2017). It suggests people’s desire for the stable framework of some ongoing relationship in which the individuals share a mutual concern for each other (DeWall and Bushman, 2011). That’s to say, when people are recognized and accepted by others, they can realize a feeling of stability of the relationship. Additionally, feelings of being supported and cared in stable social relationships are more likely to induce a strong sense of interpersonal security (Feeney and Collins, 2015). Hence, given the attributes of green products, the researchers purpose that the interpersonal symbolism of green products can provide safety value for consumers.

### The Influence of Social Crowding on Consumers’ Preference for Green Products

Since green products can provide consumers a sense of safety value, this study posits that social crowding will increase consumers’ preference for green products. A key outcome of social crowding is that it leads to violations of personal space (Puzakova and Kwak, 2017), which can be aversive and lead to greater anxiety and feelings of threat (Hall, 1966). Specifically, there needs certain space to maintain a comfortable interpersonal distance in daily activities. While under social crowding, interpersonal distance is greatly reduced, which will lead to the feeling of invasion of private space and personal territory, and then decrease the individual’s sense of control (Hall, 1966). In addition, unwanted eye contact, accidental touching, and interrupted conversations are unavoidable in crowded situations (Wei et al., 2019). Such unavoidable social interactions may distract the individual or may create group maintenance behaviors that prevent the individual from attaining his or her personal goals and thus act as a threat to the individual’s freedom (Wei et al., 2019). If individuals feel that any of their free behaviors, in which they can engage at any moment or in the future, is eliminated or threatened with elimination, the motivational state of psychological reactance will be aroused (Rosenberg and Siegel, 2021). In other words, the human psyche attempts to maintain stable levels of psychological assets related to the self, such as self-esteem, belongingness, feelings of power, and feelings of control over one’s environment (Mandel et al., 2017). So when personal space is violated, our defense system, which has evolved to deal with threats to physical survival, is likely to be activated (Lang and Bradley, 2008). Regarding the definition of safety (Maslow, 1943), the sense of control is a sense of safety. That is, under social crowding, individual safety needs will be activated to take some actions to decrease this uncontrolled feeling.

The threat of social crowding to personal space will increase people’s demand for products with safety attributes. Consumption provides significant psychological value beyond the mere functional utility offered by products and services (Mandel et al., 2017). Prior research on compensatory consumption suggests that when individuals are faced with threats that cause deficiencies in psychological resources, they offset these deficiencies with products that offer the deficient resource in a symbolic form (Han et al., 2019). For instance, researchers posited that consumers who were wake about their intelligence were more likely to choose products that associated with intelligence to help restore a momentarily shaken self-view (Gao et al., 2009). Hence, it can be said that consumers with safety needs will tend to choose products that offer a sense of safety. However, since both the functional attributes and interpersonal symbolism of green products can offer safety value to consumers, this research predicts consumers with safety needs will tend to choose green products.

Following this reasoning, this study believes that social crowding can increase consumers’ preference for green products by activating people’s need for safety. Specifically, when the degree of social crowding is higher, the private space is more likely to be violated, and hence consumers’ safety needs are more likely to be activated. However, because of the green product’s attribute of providing safety value, choosing it can relieve negative feelings caused by social crowding. Thus, social crowding increases consumers’ preference for green products.

H1: Social crowding increases consumers’ preference for green products.
H2: Safety needs mediate the relationship between social crowding and consumers’ preference for green products.

### The Moderating Effect of Extraversion–Introversion Personality Trait

Extraversion–introversion is generally defined along a “continuum” (Itani et al., 2020). Extraverted people have a friendly, intimate style of interaction, have a desire to be with other people (i.e., are sociable), relish sheer quantity of social stimulation, easily take charge, and make up their minds (Costa and McCrae, 2013). Introversion refers to a person’s tendency to be quiet, shy, inwardly focused, timid and reserved (Walker, 2020). Further, the extravert is pictured as moving toward objects in the environment, and the introvert is moving away from them (Kumar, 2020; Rahayu, 2020).

Social behaviors are varied in people with different personalities. For example, researchers examine the influential role of extraversion–introversion as a key personality trait in driving customers to engage or not engage with restaurants (Itani et al., 2020). Relatedly, extraverted customers are talkative, sociable, outgoing, and enthusiastic (Viswanathan et al., 2018). An intriguing question is whether introverts and extraverts behave differently under social crowding.

Previous research posits that certain personality characteristics and social situations are related to the physical distance that a person attempts to maintain between himself and another person (Keshmiri et al., 2019; Abdelrahman, 2020). For extraverts, since they tend to pay more attention to external necessities (Namazian and Mehdipour, 2013), they prefer to engage in more social interaction (Walker, 2020) and to seek and attract more social attention (Chiaburu et al., 2015). Furthermore, they are not shy when interacting with strangers and strive for excitement and pleasure of attention from large groups of individuals whom they are unfamiliar with (Kabadayi et al., 2014). So they are eager to break the boundaries with strangers. Additionally, they allow people to approach personal space more closely than do the introverts (Kabadayi et al., 2014) in social interaction, so their sense of boundary with others is very weak. Hence, when extraverts are under social interaction, they will be actively involved in the interaction with other people since they don’t have a strong demand for private space.

Introverts, by contrast, who stress more about their subjective values (Namazian and Mehdipour, 2013), are inclined to spend more time alone and, when they do socialize, tend to prefer more intimate settings (Feiler and Kleinbaum, 2015). Additionally, introverts typically have much lower levels of uncontrolled interactions with others, and higher levels of stress, as well as lower levels of motivation and concentration, significantly impact others (Wadu Mesthrige and Chiang, 2019). That is, an introvert might easily feel crowded in a situation that an extravert finds socially enjoyable (Zelenski et al., 2021). Thus, introverts have a strong sense of boundary with strangers, which will lead to greater physical distancing in social interactions (Park, 2015; Keem, 2017). Hence, when introverts are under social interaction, they are more likely to feel stressed and want to keep a greater distance from others since their strong demand for private space.

In conclusion, the researchers suggest that the positive relationship between social crowding and consumers’ safety needs can be moderated by extraversion–introversion personality trait. Specifically, for extraverts, since they focus more on the outside world and enjoy participating in social interaction and establishing relationships with others, they have a weak sense of boundary with strangers and thus only need a small personal distance. Therefore, in the case of social crowding, extraverts’ personal space is less likely to be invaded, so they will not feel crowded, and their safety needs will not be activated, either. Hence we can say that when consumers are extraverted, social crowding won’t affect their purchasing of green products. For introverts, by contrast, since they pay more attention to their inner landscape, feel pressure in social interaction, and desire less interaction with others, they have a strong sense of boundary with strangers and want to keep a larger distance from others in social interaction. Therefore, under social crowding, the introverts’ personal space will be invaded easily, and they are more likely to feel crowded, so their safety needs are more likely to be activated. That is, when consumers are introverted, social crowding is more likely to increase their preference for green products.

H3: The positive relationship between social crowding and consumers’ preference for green products is more likely to occur for introverted consumers rather than extraverted consumers.

## Materials and Methods

### Study 1

The purpose of study 1 is to test H1 that social crowding increases consumers’ preference for green products.

#### Participants and Design

Based on the calculation method adopted by Cohen (1977) (the Effect size *f* = 0.25 and the expected Power = 0.80), the researchers determined sample sizes of 158 by G*Power 3.1 software. One hundred and eighty participants were recruited in exchange for 10 RMB to complete a task. The study used a between-subjects design (social crowding: high vs. low vs. number control). Participants were randomly assigned to one of the three conditions. The sample size was (*N* = 167, *M_*age*_* = 25.80, *SD_*age*_* = 4.38, age range: 19–36, female 47.90%), and each group was (n*_*high social crowding*_* = 57, n*_*low social crowding*_* = 54, n*_*number control*_* = 56).

#### Stimuli and Procedure

**FIGURE 1:**
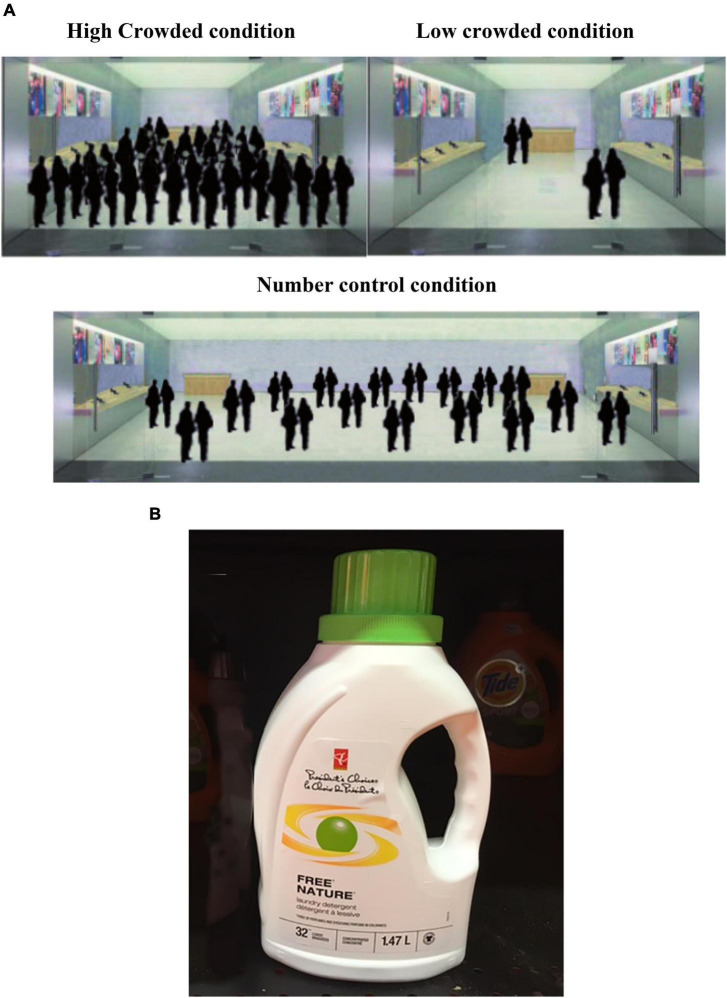
**(A)** The three pictures were converted from a real scene of the store, and the actual people were replaced by human silhouettes to protect human identity. The three pictures of the store represented three different levels of crowding. **(B)** This is a bottle of laundry detergent, which is green, natural, mild, anti sensitive, low-carbon, and environmentally friendly, with stronger decontamination ability.

A pre-test was conducted to examine the manipulation of laundry detergent used in study 1 was considered as a green product. 38 participants were recruited online (*M*_*age*_ = 25.34, *SD* = 3.96, age range 20–34, female 55.26%). All participants were presented with the image of a bottle of laundry detergent used in study 1. The image was adapted from materials used by Dixon and Mikolon (2021) (see Figure 1). The manipulation text under the image is “This is a bottle of laundry detergent, which is green, natural, mild, anti-sensitive, low-carbon and environmentally friendly, with stronger decontamination ability.” After reading the manipulation, the participants were required to answer, “Do you think it’s a green product?” The researchers then counted the percentage of participants who thought the detergent was a green product. The results showed that all the participants thought the product was a green product. The results confirmed our manipulation of green products in study 1.

In the main study, all participants were told that the purpose of this study was a survey of new products. On the manipulation of social crowding, this study referred to the study of O’Guinn et al. (2015). All participants were randomly assigned to three groups (high social crowding vs. low social crowding vs. number control). Each participant was first shown a picture of the store. They were told the picture was converted from a real scene of the store, and the actual people were replaced by human silhouettes to protect human identity. The three pictures of the store represented three different levels of crowding. Among them, participants in the high social crowding group were shown the first picture (containing 35 people), which represented a high social crowding condition. The second picture (containing 4 people), which represented low social crowding condition, was shown to participants in low social crowding group. To exclude the influence of population size, the researchers set a number control condition that the population was the same but the space was three times as large as the high crowding condition (see Figure 1).

The dependent variable was the preference for green products. All participants were then presented with the image of a bottle of laundry detergent. After reading the manipulation under the image, participants were asked to indicate their preference for the product (7-point scale, 1: *Very Unlikely*; 7: *Very Likely*).

Then they also rated on their perceived consumer effectiveness (PCE) [(α = 0.79), adapted from Roberts, 1996] and environmental concern (EC) [(α = 0.84], adapted from Roberts, 1996] with 7-point scales (1: *Strongly Disagree*; 7: *Strongly Agree*), and their current mood state [18-item (α = 0.82)] using Brief Mood Introspection Scale (BMIS; Mayer and Gaschke, 1988; 1: *Not at all;* 5: *Extremely*). Finally, participants were asked to provide ratings on their perceived crowding (adopted by Machleit et al., 1994) (1*: Strongly Disagree;* 7*: Strongly Agree*) of the store, and answered whether they thought the laundry detergent was a green product. Then they reported whether their ratings were based on their past shopping experience, guessed the purpose of this study, and completed some filler task questions about their demographic information, including gender, age, ethnicity, and income.

#### Results and Discussion

##### Manipulation Check

Thirteen participants’ preference for products depended on the past shopping experience, and no participants guessed the real purpose of the study. All the participants thought the product was a green product. Participants in high social crowding group perceived more crowded than those in low social crowding group and control group [*F*(2, 164) = 120.21, *P* < 0.001, *M_*high social crowding*_* = 5.07, *SD* = 1.05, *M_*low social crowding*_* = 2.69, *SD* = 0.97, *M_*number control*_* = 2.57, *SD* = 0.87].

There was no significant difference among the three groups in PCE [*F*(2, 164) = 0.48, *P* = 0.618, *M_*high social crowding*_* = 4.33, *SD* = 0.65, *M_*low social crowding*_* = 4.43, *SD* = 0.60, *M_*number control*_* = 4.32, *SD* = 0.75], EC [*F*(2, 164) = 0.18, *P* = 0.840, *M_*high social crowding*_* = *4.23, SD* = *0.81, M_*low social crowding*_* = *4.30, SD* = *0.68, M_*number control*_* = *4.23, SD* = *0.78]*, and current mood state [*F*(2, 164) = 2.05, *P* = 0.132, *M_*high social crowding*_* = 4.06, *SD* = 0.65, *M_*low social crowding*_* = 4.03, *SD* = 0.70, *M_*number control*_* = 3.82, *SD* = 0.67].

##### The Preference for Product

**FIGURE 2:**
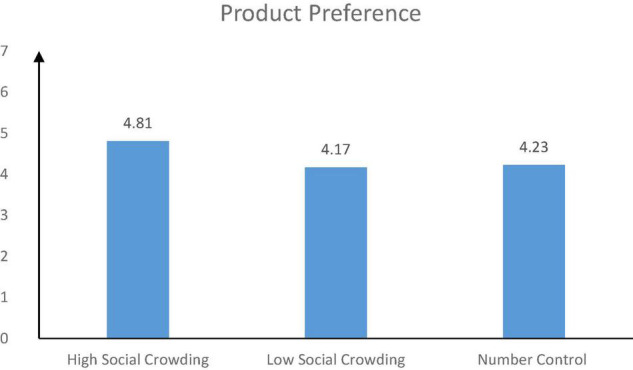
The results of product preference.

The results showed that there was a significant difference in the preference for green products among the three groups [*F*(2, 164) = 8.04, *p* < 0.001]. The participants in the high social crowding group (*M_*high social crowding*_* = 4.81, *SD* = 0.95) had a greater preference for green product than those in the low social crowding group [*M*_*low social crowding*_ = 4.17, *SD* = 1.00, *t*(164) = 3.62, *p* < 0.001] and the control group [*M*_*number control*_ = 4.23, *SD* = 0.83, *t*(164) = 0.37, *p* < 0.001], and the product preference of the control group was greater than that of the low social crowding group [*t*(164) = 3.28, *p* < 0.001] (see Figure 2). The results provided the evidence for H1.

Study 1 showed that social crowding significantly affected consumers’ preference for green products. Under the condition of high social crowding (vs. low social crowding), consumers were more likely to prefer green products. This effect was not affected by population size. In other words, in the context of high social crowding, people were more likely to choose and purchase green products with attributes of environmental protection.

Study 1 manipulated the degree of social crowding in the laboratory, but the researchers were not sure about consumers’ choices in a real-life crowded environment. Therefore, study 2 was in a real-life crowded situation, and used another kind of product, shampoo, to increase the robustness of the results.

### Study 2

The purpose of this study is to replicate the results of study 1 in a real-life crowded situation, and also test H2, in which safety needs mediate the relationship between social crowding and consumers’ preference for green products.

#### Participants and Design

Based on the calculation method adopted by Cohen (1977) (the Effect size *d* = 0.5 and the expected Power = 0.80), the researchers determined sample sizes of 128 by G*Power 3.1 software. One hundred and fifty participants were recruited in a university to participate in study 2 to complete a brief survey about mobile phones in exchange for a bottle of shampoo. The study used a between-subjects design (social crowding: high vs. low). Participants were randomly assigned to one of the two conditions. The sample size was (*N* = 135, *M_*age*_* = 24.34, *SD_*age*_* = 3.46, age range: 19–33, female 46.67%), and each group was (n*_*high social crowding*_* = 68, n*_*low social crowding*_* = 67).

#### Stimuli and Procedure

**FIGURE 3:**
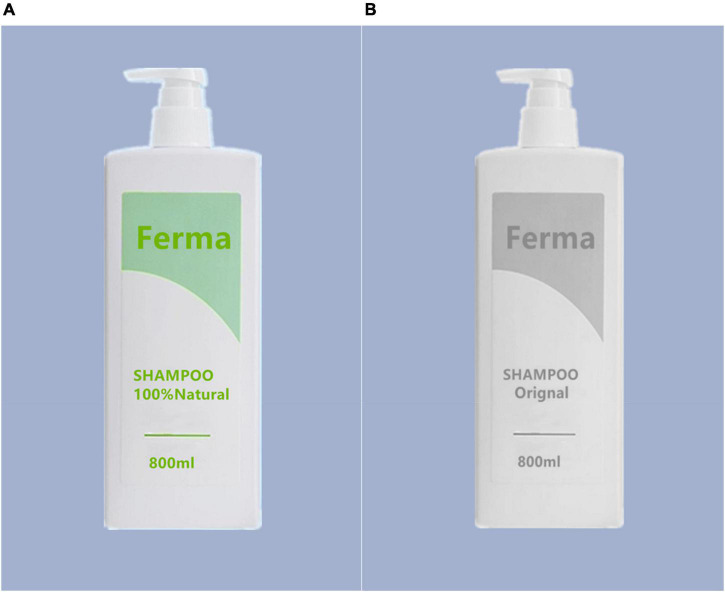
**(A)** “Ferma” is a very famous natural shampoo product. It extracts pure natural plant essence without addition, safe and reliable. It can deeply clean the scalp, solve a variety of hair problems, and protect your soft hair. **(B)** “Ferma” is a very famous original shampoo product. Its texture is mild and its foam is rich. It can deeply clean the scalp, solve a variety of hair problems, and protect your soft hair.

A pre-test was conducted to examine the manipulation of the attributes of shampoo used in study 2. The researchers created a fictitious brand of shampoo, “Ferma.” Seventy five participants were recruited online (*M*_*age*_ = 24.64, *SD* = 3.28, age range 20–35, female 50.67%). All participants were randomly assigned to two groups (green product vs. common product). Participants in each group read the corresponding manipulation about shampoo and related pictures (see Figure 3). The green product group was presented with the image of a bottle of green shampoo. The manipulation text under the image is “Ferma” is a very famous natural shampoo product. It extracts pure natural plant essence without addition, safe and reliable. It can deeply clean the scalp, solve a variety of hair problems, and protect your soft hair.” The common product group was presented with the image of a bottle of common shampoo. The manipulation text under the image is “Ferma” is a very famous original shampoo product. Its texture is mild and its foam is rich. It can deeply clean the scalp, solve a variety of hair problems, and protect your soft hair.”

After reading the manipulation, the participants were required to answer, “Do you think it’s a green product?” (1–7 point scale, 1 = I think it is surely a common product, 7 = I think it is surely a green product). The results showed that participants in green product group reported higher results than those in common product group [*M*_*green product*_ = 5.31, *SD* = 0.92, *M*_*common product*_ = 2.50, *SD* = 0.85, *t*(73) = 13.71, *P* < 0.001]. The results confirmed our manipulation of product attributes in study 2.

In the main study, all participants were told that the purpose of this study was a survey of mobile phones. After completing it, they would be rewarded with a bottle of shampoo. Each participant was first asked to fill in the survey about their mobile phone brand, service life, and factors affecting their purchase of a mobile phone. The survey had nothing to do with the real purpose of this study.

After completing the survey, the participants in each group (high social crowding vs. low social crowding) were taken into a corresponding room. In terms of the manipulation of social crowding, participants in the high social crowding group were taken into a high crowded room (15 square meters, 20–23 fake participants here), while participants in the low social crowding group were taken into a low crowded room (15 square meters, 4–6 fake participants here). There were two kinds of shampoo information on the computer, green or common (see Figure 3). A brief introduction was under the product correspondingly. Participants were told to choose a bottle of shampoo as a reward.

Then they were invited to fill in a brief survey, including their safety needs [(α = 0.89)], adapted from Neal et al., 2000) (1*: Strongly Disagree;* 7*: Strongly Agree*), their willingness to choose the shampoo product (1: I am inclined to choose the common shampoo; 7: I am inclined to choose the green shampoo). Then, all participants were asked to provide ratings on their perceived crowding of the room. They also rated on their PCE, EC, and current mood state. Finally, participants reported whether their choices were based on their past shopping experience and guessed the purpose of this study. The demographic information of participants such as age, gender, race, and income was collected.

#### Results and Discussion

##### Manipulation Check

Fifteen participants’ preferences for the product depended on the past shopping experience, and no participants guessed the real purpose of the study. Participants in high social crowding group perceived more crowded than those in low social crowding group [*M_*high social crowding*_* = 5.04, *SD* = 1.01, *M_*low social crowding*_* = 2.64, *SD* = 0.98, *t*(133) = 13.99, *p* < 0.001].

There was no significant difference between the two groups in PCE [*M_*high social crowding*_* = 4.23, *SD* = 0.62, *M_*low social crowding*_* = 4.10, *SD* = 0.71, *t*(133) = 1.08, *p* = 0.283], EC [*M_*high social crowding*_* = 4.15, *SD* = 0.79, *M_*low social crowding*_* = 4.16, *SD* = 0.70, *t*(133) = 0.10, *p* = 0.921], and current mood state [*M_*high social crowding*_* = 3.97, *SD* = 0.64, *M_*low social crowding*_* = 3.97, *SD* = 0.66, *t*(133) = 0.04, *p* = 0.972].

##### The Preference for Product

Participants in high social crowding group exhibited a higher willingness of choosing green product than did those in low social crowding group [*M_*high social crowding*_* = 4.85, *SD* = 0.92, *M_*low social crowding*_* = 4.37, *SD* = 0.85, *t*(133) = 3.15, *p* = 0.002].

##### Safety Needs

The results showed that there was a significant difference in safety needs between the two groups [*t*(133) = 3.59, *P* < 0.001]. The safety needs of participants in the high social crowding group were higher than those in the low social crowding group [*M_*high social crowding*_* = 4.35, *SD* = 0.84, *M_*low social crowding*_* = 3.85, *SD* = 0.78, *t*(133) = 3.59, *p* < 0.001].

##### The Analysis of the Mediating Effect

**FIGURE 4:**
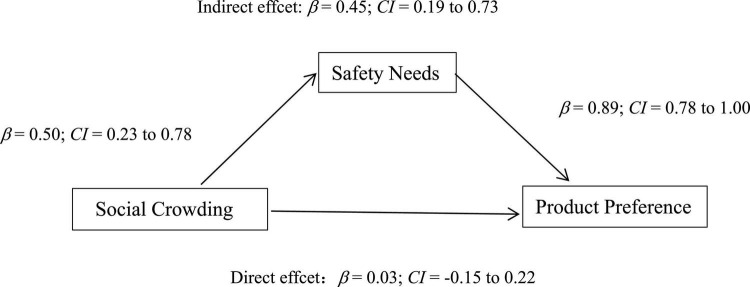
The mediating effect of safety needs.

A bootstrapping analysis (PROCESS Model 4, Hayes, 2013) was used to analyze the mediating role of safety needs between social crowding and product preference. Social crowding (high social crowding vs. low social crowding) was taken as the independent variable, product preference as the dependent variable, and safety needs as the mediating variable. The results showed that safety needs mediated the influence of high social crowding on product preference (95% confidence interval β = 0.45; CI = 0.19–0.73), which confirmed H1 (see Figure 4).

Study 2 illustrated that safety needs mediated the relationship between social crowding and consumers’ preference for green products. High social crowding (vs. low social crowding) could threaten consumers’ private space and elicit the need for safety. Therefore, consumers tended to choose green products to meet the need for safety.

### Study 3

Study 3 aims to further clarify the boundary of the main effect, that is, the impact of social crowding on the preference for products is only significant in green products.

#### Participants and Design

Based on the calculation method adopted by Cohen (1977) (the Effect size *f* = 0.25 and the expected Power = 0.80), the researchers determined sample sizes of 179 by G*Power 3.1 software. 210 participants were recruited in exchange for 10 RMB to complete a task. The study used a between-subjects design 2 (social crowding: high vs. low) × 2 (product: green vs. common). Participants were randomly assigned to one of the four conditions. The sample size was (*N* = 191, *M_*age*_* = 24.59, *SD_*age*_* = 4.16, age range: 18–37, female 51.83%), and each group was (n*_*high social crowding green product*_* = 48, n*_*high social crowding common product*_* = 48, n*_*low social crowding green product*_* = 46, n*_*low social crowding common product*_* = 49).

#### Stimuli and Procedure

**FIGURE 5:**
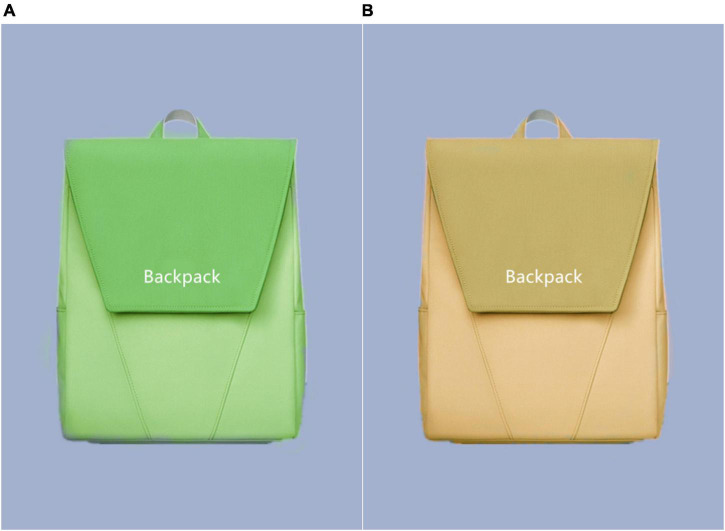
**(A)** This is a green backpack, which is made from environmentally friendly materials, simple and versatile. **(B)** This is a common backpack, which is made of polyester fiber, simple and versatile.

A pre-test was conducted to examine the manipulation of the attributes of backpack used in study 3. 82 participants were recruited online (*M*_*age*_ = 22.72, *SD* = 2.04, age range 18–27, female 48.78%). All participants were randomly assigned to two groups (green product vs. common product). Participants in each group read the corresponding manipulation about the backpack and related pictures. The green product group was presented with the image of a green backpack. The manipulation text under the image is “This is a green backpack, which is made from environmentally friendly materials, simple and versatile.” The common product group was presented with the image of a common backpack (see Figure 5). The manipulation text under the image is “This is a common backpack, which is made of polyester fiber, simple and versatile.”

After reading the manipulation, the participants were required to answer, “Do you think it’s a green product?” (7 point scale, 1 = It is surely a common product, 7 = It is surely a green product). The results showed that participants in green product group evaluated product attributes significantly higher than those in common product group [*M*_*green product*_ = 5.10, *SD* = 1.02, *M*_*common product*_ = 2.83, *SD* = 0.83, *t*(80) = 11.03, *P* < 0.001]. The results confirmed our manipulation of product attributes in study 3.

In the main study, all participants were told that the purpose of this study was a survey of new products. The manipulation of social crowding was similar to study 1. The only difference was that there were only two conditions of social crowding (high social crowding vs. low social crowding).

After that, the participants in each group (high social crowding vs. low social crowding) were presented with product images correspondingly. In terms of the manipulation of product attributes, participants in the green product group received the information of green backpack, while participants in the common product group received the information of common backpack.

Participants then were asked to indicate their preference for the product and safety needs. They also rated on their PCE, EC, and current mood state. Finally, participants were asked to provide ratings on their perceived crowding of the store and the product attribution. Then they reported whether their choices were based on their past shopping experience, guessed the purpose of this study, and completed some filler task questions about their demographic information, including gender, age, ethnicity, and income.

#### Results and Discussion

##### Manipulation Check

Nineteen participants’ preferences for product depended on past shopping experience, and no participants guessed the real purpose of the study. Participants in high social crowding group perceived more crowded than those in low social crowding group [*M_*high social crowding*_* = 4.14, *SD* = 1.62, *M_*low social crowding*_* = 4.00, *SD* = 1.55, *t*(189) = 0.60, *p* = 0.547]. Participants in green product group evaluated product attributes significantly higher than those in common product group [*M*_*green product*_ = 5.27, *SD* = 0.89, *M*_*common product*_ = 2.54, *SD* = 0.88, *t*(189) = 21.28, *P* < 0.001].

There was no significant difference between the two groups in PCE [*M_*high social crowding*_* = 4.04, *SD* = 0.63, *M_*low social crowding*_* = 4.11, *SD* = 0.64, *t*(189) = 0.73, *p* = 0.469], EC [*M_*high social crowding*_* = 4.00, *SD* = 0.73, *M_*low social crowding*_* = 4.09, *SD* = 0.71, *t*(189) = 0.87, *p* = 0.386], and current mood state [*M_*high social crowding*_* = 4.00, *SD* = 0.61, *M_*low social crowding*_* = 3.97, *SD* = 0.72, *t*(189) = 0.32, *p* = 0.748].

##### Safety Needs

The results showed that the interaction between social crowding (high social crowding vs. low social crowding) and product type (green product vs. common product) didn’t affect safety needs (*F* = 0.18, *P* = 0.673). In the context of common product, there was significant difference in the safety needs between the two groups [*M_*high social crowding*_* = *4.27, SD* = *0.84, M_*low social crowding*_* = *3.84, SD* = *0.99, t*(95) = 2.33, *p* = 0.022]. However, in the context of green products, there was also a significant difference in safety needs between the two groups. The safety needs of high social crowding group was significantly higher than that of low social crowding group [*M_*high social crowding*_* = 4.42, *SD* = 0.92, *M_*low social crowding*_* = 3.87, *SD* = 0.93, *t*(92) = 2.86, *p* = 0.005].

##### The Preference for Product

**FIGURE 6:**
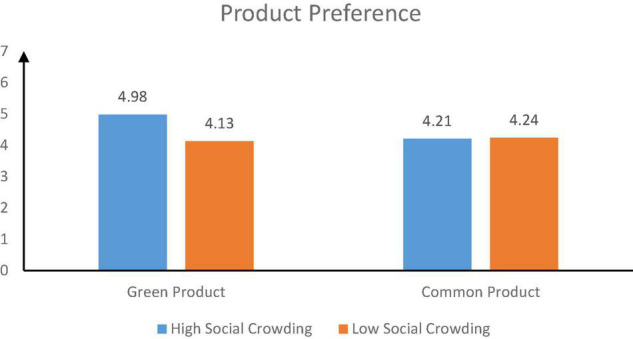
The moderating effect of product type.

The results showed that the interaction between social crowding (high social crowding vs. low social crowding) and product type (green product vs. common product) significantly affected product preference (*F* = 9.76, *P* = 0.002). In the context of common products, there was no significant difference in product preference between high social crowding group and low social crowding group [*M_*high social crowding*_* = 4.21, *SD* = 0.99, *M_*low social crowding*_* = 4.24, *SD* = 0.83, *t*(95) = 0.20, *p* = 0.844]. In the context of green products, there were significant differences in product preferences between the two groups. The product preference of the high social crowding group was significantly greater than that of the low social crowding group [*M_*high social crowding*_* = 4.98, *SD* = 1.00, *M_*low social crowding*_* = 4.13, *SD* = 1.09, *t*(92) = 3.94, *p* < 0.001] (see Figure 6).

##### Moderated Mediating Effect

Taking social crowding (high social crowding vs. low social crowding) as the independent variable, product preference as the dependent variable, safety needs as the mediating variable, and product type as the moderating variable, bootstrapping (PROCESS Model 14, Hayes, 2013) was used to analyze the mediating role of product type (green product vs. common product). Social crowding significantly affected safety needs (95% confidence interval = 0.49; CI = 0.23–0.75). The interaction between social crowding and product type significantly affected the product preference (95% confidence interval = 1.05; CI = 0.82–1.27). When the product was green, social crowding significantly affected the product preference via safety needs (conditional indirect effect, 95% confidence interval = 0.51; CI = 0.25–0.81). When the product was common, the indirect effect of social crowding on product preference was not significant (conditional indirect effect, 95% confidence interval = –0.01; CI = –0.15 to 0.12). In conclusion, the interaction between social crowding and product type significantly affected product preference (95% confidence interval = 0.51; CI = 0.25–0.86).

Study 3 found that product type (green products vs. common products) moderated the relationship between social crowding and consumers’ preference. Social crowding significantly influenced consumers’ preference for a product only when the product was a green product. Consumers in high social crowding had higher preferences for green products than those in low social crowding. However, when the product was a common product, there was no significant difference between consumers in high and low social crowding conditions.

### Study 4

The purpose of this study was to test H3, in which the researchers proposed the positive relationship between social crowding and consumers’ preference for green products was more likely to occur for introverted consumers rather than extraverted consumers. The researchers used another way, written scenario induction, to manipulate the degree of social crowding.

#### Participants and Design

Based on the calculation method adopted by Cohen (1977) (the effect size *f* = 0.25 and the expected power = 0.80), the researchers determined sample sizes of 179 by G*Power 3.1 software. Three hundred participants were recruited in exchange for 10 RMB to complete a task. The study used a between-subjects design 2 (social crowding: high vs. low) × 2 (personality traits: introversion vs. extraversion). Participants were first asked to complete the extraversion–introversion measure using Introversion Scale [12-item (α = 0.8), McCroskey, 2007] on a 5-point Likert scale (1: *Strongly Disagree*; 5: *Strongly Agree*), which divided them into introversion group and extraversion group (scoring above 48: highly introverted; scoring below 24: highly extraverted). Then they were randomly allocated to two groups (high social crowding vs. low social crowding). The sample size was (*N* = 184, *M_*age*_* = 24.55, *SD_*age*_* = 4.15, age range: 18–37, female 51.63%), and each group was (n*_*high social crowding introversion*_* = 47, n*_*high social crowding extraversion*_* = 44, n*_*low social crowding introversion*_* = 46, n*_*low social crowding extraversion*_* = 47).

#### Stimuli and Procedure

A pre-test was conducted to examine the manipulation of the social crowding in study 4 increased feelings of crowding. The manipulation was adapted from the study of Coskun et al. (2019). 85 participants were recruited online (*M*_*age*_ = 24.53, *SD* = 4.49, age range 18–35, female 50.59%). All participants were randomly assigned to two groups (high social crowding vs. low social crowding). Participants in each group read the corresponding text. The participants in the high social crowding group read the manipulation emphasizing feelings of high social crowding, “Imagine that it is weekend and you are strolling around in a shopping mall. When passing a store, you decide to go in and browse. You enter and notice that the store is very crowded and filled with many people. Because of the large number of other customers, it is really hard to move smoothly through the store. Other people often bump into you.” The participants in the low social crowding group read the manipulation emphasizing feelings of low social crowding, “Imagine that it is weekend and you are strolling around in a shopping mall. When passing a store, you decide to go in and browse. You enter and notice that the store is fairly empty with only a few customers. Because of the lack of other customers, it is really easy to move smoothly through the store. Other people do not bump into you.” After that, participants provided ratings assessing their feelings of crowding (adopted by Machleit et al., 1994) (1*: Strongly Disagree;* 7*: Strongly Agree*). The results showed that participants in the high social crowding group rated the degree of crowding significantly higher than those in the low social crowding group [*M_*high*_ _*social crowding*_* = 5.07, *SD* = 1.05, *M_*low*_ _*social crowding*_* = 2.38, *SD* = 0.93, *t*(83) = 12.45, *P* < 0.001]. The results confirmed our manipulation of social crowding in study 4.

In the main study, all participants were told that the purpose of this study was a survey of new products. Participants in each group were asked to read the corresponding written scenario as the pre-test.

After the reading task, the participants read the manipulation about the shampoo and related picture (the same as materials used in study 2) and then were asked to indicate their preference for the product, green or common (7-point scale, 1*: Common product;* 7*: Green product*), and safety needs.

They also rated on their PCE, EC, and current mood state. Finally, participants were asked to provide ratings on their perceived crowding of the store. Then they reported whether their choices were based on their past shopping experience, guessed the purpose of this study, and completed some filler task questions about their demographic information, including gender, age, ethnicity, and income.

#### Results and Discussion

##### Manipulation check

One hundered and three participants (scoring below 48 and above 24 in the extraversion–introversion measure) were deleted. 13 participants’ preferences for the product depended on the past shopping experience, and no participants guessed the real purpose of the study. Participants in high social crowding group perceived more crowded than those in low social crowding group [*M_*high social crowding*_* = 5.27, *SD* = 1.05, *M_*low social crowding*_* = 2.87, *SD* = 0.92, *t*(182) = 16.46, *p* < 0.001].

There was no significant difference between the two groups in PCE [*M_*high social crowding*_* = 4.14, *SD* = 0.71, *M_*low social crowding*_* = 4.10, *SD* = 0.67, *t*(182) = 0.36, *p* = 0.722], EC [*M_*high social crowding*_* = 4.02, *SD* = 0.70, *M_*low social crowding*_* = 4.05, *SD* = 0.87, *t*(182) = 0.27, *p* = 0.785], and current mood state [*M_*high social crowding*_* = 4.06, *SD* = 0.73, *M_*low social crowding*_* = 4.13, *SD* = 0.73, *t*(182) = 0.59, *p* = 0.558].

##### Safety Needs

*T*he results showed that the interaction between social crowding and introversion-extraversion personality traits significantly affected the safety needs (*F* = 5.72, *P* = 0.018). The results of simple slope test showed that when consumers were extraverted, social crowding didn’t affect their safety needs [*M_*high social crowding*_* = 3.77, *SD* = 0.77, *M_*low social crowding*_* = 3.81, *SD* = 0.97, *t*(89) = 0.19, *p* = 0.847]. However, when consumers were introverted, social crowding significantly affected their needs for safety [*M_*high social crowding*_* = 4.34, *SD* = 1.09, *M_*low social crowding*_* = 3.72, *SD* = 0.86, *t*(91) = 3.06, *p* = 0.003].

##### The Preference for Product

**FIGURE 7:**
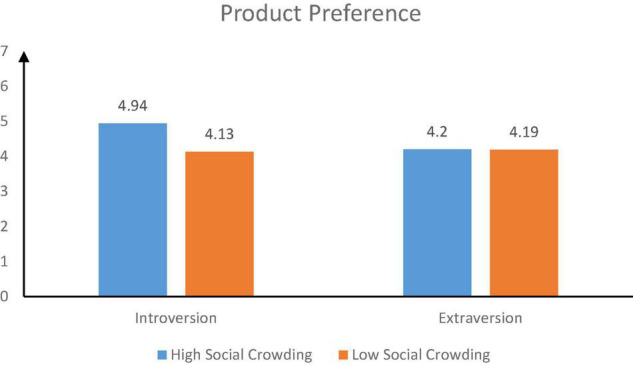
The moderating effect of personality trait.

The results showed that the interaction between social crowding and introversion-extraversion personality traits significantly affected product preference (*F* = 7.37, *P* = 0.007). The results of simple slope test showed that when consumers were extraverted, social crowding didn’t affect consumers’ product preference [*M_*high social crowding*_* = 4.20, *SD* = 1.00, *M_*low social crowding*_* = 4.19, *SD* = 1.08, *t*(89) = 0.06, *p* = 0.952]. However, when consumers were introverted, social crowding significantly affected their preference for product [*M_*high social crowding*_* = 4.94, *SD* = 1.01, *M_*low social crowding*_* = 4.13, *SD* = 0.86, *t*(91) = 4.14, *p* < 0.001] (see Figure 7). The results confirmed H3.

##### Moderated Mediating Effect

A bootstrapping (PROCESS Model 7, Hayes, 2013) was used to analyze the moderated mediating role of introversion-extraversion personality traits. The results showed that the interaction between social crowding (high social crowding vs. low social crowding) and introversion-extraversion personality traits significantly affected consumers’ safety needs (95% confidence interval β = 0.66, CI = 0.12–1.20). Meanwhile, safety needs significantly affected consumers’ product preference (95% confidence interval β = 0.94, CI = 0.86–1.01). When consumers were introverted, social crowding significantly affected their product preference via security needs (confidence interval β = 0.58; CI = 0.21–0.97). When consumers were extraverted, the indirect effect of social crowding on consumers’ product preference was not significant (95% confidence interval β = –0.03; CI = –0.39 to 0.31). In conclusion, introversion- extraversion personality traits significantly moderated the relationship between social crowding and consumers’ preference via safety needs (95% confidence interval β = 0.62, CI = 0.11–1.14).

Study 4 showed that consumers’ personality traits significantly moderated the relationship between social crowding and consumers’ preference for green products. Only when consumers were introverted, social crowding significantly affect consumers’ preference for green products. At this point, the higher the level of social crowding, the higher consumers’ preference for green products. However, when consumers were extraverted, social crowding could not affect consumers’ preference for green products.

## Discussion

By introducing the theory of social crowding from the field of sociology to the field of consumer product preference research, this research explores and demonstrates the impact of social crowding on the preference for green products and its mediating mechanism. Besides, this research also proposes and examines the moderating effect of introversion-extraversion personality traits on the above effects.

Study 1 manipulated the degree of social crowding to explore consumers’ preference for green products. The results showed that consumers prefer green products more under the condition of high social crowding than under the low. Study 2 was carried out in a real-life crowded situation to increase the reliability of the results and simultaneously proved the mediating role of safety needs in the influence of social crowding on the preference for green products. Study 3 further clarified the boundary of the main effect, that is, the impact of social crowding on the preference for products is only significant in green products. Study 4 demonstrated the moderating effect of introversion-extraversion personality traits between social crowding and green product preference. Specifically, when consumers are extraverted, social crowding won’t affect their preference for green products. When consumers are introverted, social crowding is more likely to increase their preference for green products.

### Theoretical Contributions

The theoretical contributions of this research are mainly as follows:

Firstly, this study employs social crowding in the research of green consumption, builds the positive relationship between social crowding and the willingness to buy green products, enriches the relevant research in the field of green consumption. The existing research explore the factors influencing green consumption behavior from three perspectives: individual factors, social relationship, the characteristic of brand and store. From the individual perspective, previous studies find environmental consciousness (Kim and Chung, 2011; Kautish et al., 2019), pro-social self-concept (Johnson et al., 2018), the desire for status (Griskevicius et al., 2010), cosmopolitanism, global self-identity (Khare and Kautish, 2020), terminal value and instrumental value (Kautish and Sharma, 2018, 2019b; Kautish et al., 2020), trust (Lăzăroiu et al., 2019), demographic variable (Pocol et al., 2021), are positively related to consumers’ willingness to buy green products. From the social relationship perspective, consumers’ green consumption behaviors are influenced by social cognitive framework (Johnstone and Hooper, 2016), specific social groups, and social norms (Salazar et al., 2012). From the characteristic of brand and store, green brand image (Alamsyah et al., 2020), perceived store accessibility (Chang and Watchravesringkan, 2018), availability and ease of purchase (Grimmer et al., 2016), store-related attributes of eco-fashion (Chan and Wong, 2012), retail formats (Dabija et al., 2018), and corporate social responsibility (May et al., 2021) have important impact on green consumption. As a common consumption context, social crowding exists in people’s green consumption. However, previous studies have mainly focused on individual factors, marketing characteristics and social relationship, but have not explored the impact of social crowding on consumers’ preference for green products. This study embeds social crowding into the research field of green consumption, finds that social crowding increases consumers’ preference for green products through activating safety needs. It focuses on the impact of social crowding on green consumption behavior for the first time, constructs the main effect framework in the theoretical and applied fields, and expands the relevant literature in the field of green consumption.

Secondly, the present research firstly proposes and empirically examines the mediating role of safety needs in the relationship between social crowding and consumers’ preference for green products. Safety needs is an important factor in consumer behavior field (Rydell and Kucera, 2021). Previous studies explore the role of safety needs in the relationship between epidemic disease and consumer behavior (Birtus and Lăzăroiu, 2021). For example, the threat posed by COVID-19 leads to panic buying (Hopkins and Potcovaru, 2021; Watson and Popescu, 2021). However, previous studies haven’t proposed and demonstrated the mediating role of safety needs between the relationship of social crowding and green consumption. Hence, the current study focuses on the specific social situation of social crowding and suggests the psychological origin of safety needs from the perspective of the interaction between individuals and green products. Social crowding threatens individual’s private space, activates safety needs and increases green consumption. Thus, it identifies the role of safety needs in reshaping a stable psychological environment to deal with social crowding and then establishes a mediating model between social crowding and green products preference. Therefore, this study builds the theoretical relationship between social crowding and safety needs, and meanwhile explains and connects the relationship between the implicit psychological perception of safety needs and the explicit consumption behavior variable of green products preference.

Thirdly, this paper also introduces personality trait variable, introversion-extraversion, with broader significance, to prove the role of individual personality traits under social crowding, and also expands the literature on introversion and extraversion. Previous studies are mainly on the field of psychology, discussing the personality characteristics of introverted and extraverted individuals (Maeng et al., 2013), their expression in social interaction (Costa and McCrae, 2013), and the quality of their social relationship (Kumar, 2020; Rahayu, 2020). However, few studies explore the influence of introversion and extraversion on consumer behaviors and decision-making in green consumption. Taking consumer product decision-making as the research context, this study firstly identifies the characteristics of introversion and extraversion under social crowding, and clearly defines that personality traits play a moderating role. The positive relationship between social crowding and consumers’ preference for green products is more likely to occur for introverted consumers rather than extraverted consumers. The present research proposes that due to the strong sense of boundary between introverts and others, their personal space is easy to be violated, which enhances the role of safety needs, and thus increases the green products preference effect caused by social crowding.

Finally, the current study improves the internal validity of research results through diversified manipulations on independent variable (social crowding) and dependent variable (the preference for green products). In terms of the manipulation of independent variable, previous studies mainly use picture stimuli to stimulate the participants’ perception of social crowding (O’Guinn et al., 2015; Hwang et al., 2020), and lack the collection of consumers’ responses in the real crowding situation. In the four studies, the researchers use different forms of social crowding manipulations, including stimulating participants’ perception of crowding in the laboratory by pictures (study1 and study 3) and words (study 4) and creating a real crowded situation (study 2). This study ensures the effectiveness of social crowding manipulation through diversified methods. In the manipulation of dependent variable, previous studies mostly use a single category of green products (Chen and Chiu, 2015; Brough et al., 2016; De Angelis et al., 2017), while this study uses different categories of green products in the experiment to expand the validity of the research results, including launch detergent (study 1), shapoo (Study 2 and study 4), and backpack (study 3). The results are highly consistent in all four studies. To summary, it provides strong empirical support for the relationship between social crowding and consumers’ preference for green products.

### Managerial Implications

The present research shows a meaningful combination of psychology and sociology, which can help enterprises better understand people’s green consumption patterns and behaviors in social crowding situations.

Firstly, this study explores the impact of social crowding on consumers’ green consumption. The results show that the higher the degree of social crowding, the stronger consumers’ willingness to buy green products. This study provides suggestions for enterprises to improve consumers’ positive attitudes toward green products by manipulating social crowding. For example, enterprises can manipulate consumers’ perception of crowding through words, pictures and real crowding situations.

Secondly, this study reveals the important impact of safety needs on consumers’ green consumption, and helps enterprises better understand the key role of consumer’ safety needs in green product purchase. Enterprises can enhance consumers’ willingness to buy green products by improving consumers’ safety needs.

Thirdly, this study focuses on consumer personality traits (introversion and extraversion), which is conducive to enterprises to make use of the individual factor more accurately in selling green products. Particularly, for introverted consumers, enterprises can effectively improve their positive attitudes toward green products through social crowding.

### Limitations and Future Research

The selection of sample size has some limitations to a certain extent. The main choice is the student sample, which is lack diversity. Future research can further explore whether different social groups have differences in the choice of green products.

Additionally, in the actual shopping choice, many factors affect consumers’ shopping choices. However, this research only selected some variables, for other possible factors such as price, product location, ambient temperature when shopping, etc., haven’t been taken into consideration, so further research can explore these factors that affect consumers’ preference.

Finally, in addition to the consumption of green products, some other consumer psychology and behaviors may also be affected by safety needs. Therefore, future research can further explore the individual’s preference for other objects.

## Data Availability Statement

The original contributions presented in the study are included in the article/Supplementary Material, further inquiries can be directed to the corresponding author/s.

## Ethics Statement

The studies involving human participants were reviewed and approved by the Ethics Review Committee of Center for Psychological Science and Health Research, China University of Geosciences. The patients/participants provided their written informed consent to participate in this study.

## Author Contributions

FW was responsible for the logical reasoning of the research topic. WL was responsible for experimental materials and data. GC was responsible for collecting literature. All authors contributed to the article and approved the submitted version.

## Conflict of Interest

The authors declare that the research was conducted in the absence of any commercial or financial relationships that could be construed as a potential conflict of interest.

## Publisher’s Note

All claims expressed in this article are solely those of the authors and do not necessarily represent those of their affiliated organizations, or those of the publisher, the editors and the reviewers. Any product that may be evaluated in this article, or claim that may be made by its manufacturer, is not guaranteed or endorsed by the publisher.
